# Quercetin inhibits caspase-1-dependent macrophage pyroptosis in experimental folic acid nephropathy

**DOI:** 10.1186/s13020-024-00885-2

**Published:** 2024-01-16

**Authors:** Xianli Gao, Caiyun Guo, Wenjun Li, Yingdong Deng, Wenjun Ning, Jiaqi Xie, Xiaoying Zhan, Youling Fan, Hongtao Chen, Zengping Huang, Jun Zhou

**Affiliations:** 1 Department of Anesthesiology, The Third Affiliated Hospital of Southern Medical University, Guangzhou, China; 2 Department of Anesthesiology, Shenzhen Traditional Chinese Medicine Hospital, Shenzhen, China; 3 Department of Anesthesiology, The First People’s Hospital of Kashgar, Xinjiang, China; 4 Department of Anesthesiology, The Second People’s Hospital of Panyu, Guangzhou, China; 5 Department of Anesthesiology, Guangzhou Eighth People’s Hospital, Guangzhou Medical University, Guangzhou, China

**Keywords:** Quercetin, Caspase-1, Macrophage, Pyroptosis, Folic acid

## Abstract

**Background:**

The role of pyroptosis in kidney disease is limited and incomplete. Quercetin, a flavonoid compound present in a variety of fruits, vegetables, and plants, has shown antioxidant and anti-inflammatory properties. This study was designed to validate the importance of pyroptosis in an experimental model of folic acid nephropathy and to explore the effect of quercetin in protecting against pyroptosis.

**Methods:**

Gene set enrichment analysis (GSEA) and weighted gene co-expression network analysis (WGCNA) were used to establish the correlation between pyroptosis and folic acid nephropathy. Immune cell infiltration, network pharmacology and single-cell RNA sequencing analysis were utilized to ascertain the specific target of quercetin in relation to pyroptosis. Finally, quercetin’s role was verified in vivo and in vitro.

**Results:**

The GSEA analysis revealed a significant correlation between pyroptosis and folic acid nephropathy (NES = 1.764, *P* = 0.004). The hub genes identified through WGCNA were closely associated with inflammation. Molecular docking demonstrated a strong binding affinity between quercetin and caspase-1, a protein known to be involved in macrophage function, as confirmed by immune cell infiltration and single-cell analysis. Quercetin demonstrated a significant amelioration of kidney injury and reduction in macrophage infiltration in the animal model. Furthermore, quercetin exhibited a significant inhibition of caspase-1 expression, subsequently leading to the inhibition of pro-inflammatory cytokines expression, such as IL-1β, IL-18, TNF-α, and IL-6. The inhibitory effect of quercetin on macrophage pyroptosis was also confirmed in RAW264.7 cells.

**Conclusion:**

This study contributes substantial evidence to support the significant role of pyroptosis in the development of folic acid nephropathy, and highlights the ability of quercetin to downregulate caspase-1 in macrophages as a protective mechanism against pyroptosis.

**Supplementary Information:**

The online version contains supplementary material available at 10.1186/s13020-024-00885-2.

## Introduction

Folic acid (FA) is a crucial factor in cellular growth and division, as it plays an essential role in the synthesis of DNA and RNA. Adequate intake of FA before and during early pregnancy significantly reduces the risk of neural tube defects [1]. FA supplementation may be prescribed to prevent various diseases, such as stroke [2], cardiovascular disease [3], and depression [4]. Nevertheless, excessive FA consumption can result in serious complications, including kidney injury [5].

Pyroptosis, a form of programmed cell death, assumes a pivotal role in diverse kidney diseases [6]. Cellular swelling and rupture occur during the pyroptotic process, releasing intracellular contents and eliciting an immune response. Consequently, this cascade engenders cellular demise and inflammation, contributing to kidney injury. Studies have demonstrated that the targeting of inflammasomes and caspase-1 (CASP-1) can mitigate the inflammatory response and cellular death associated with pyroptosis, thereby reducing kidney injury [7, 8]. However, the specific role of pyroptosis in FA-induced renal injury is unclear. It has clinical application prospects to seek drugs to reduce renal injury by reducing cell pyrodeath.

**Fig. 1:**
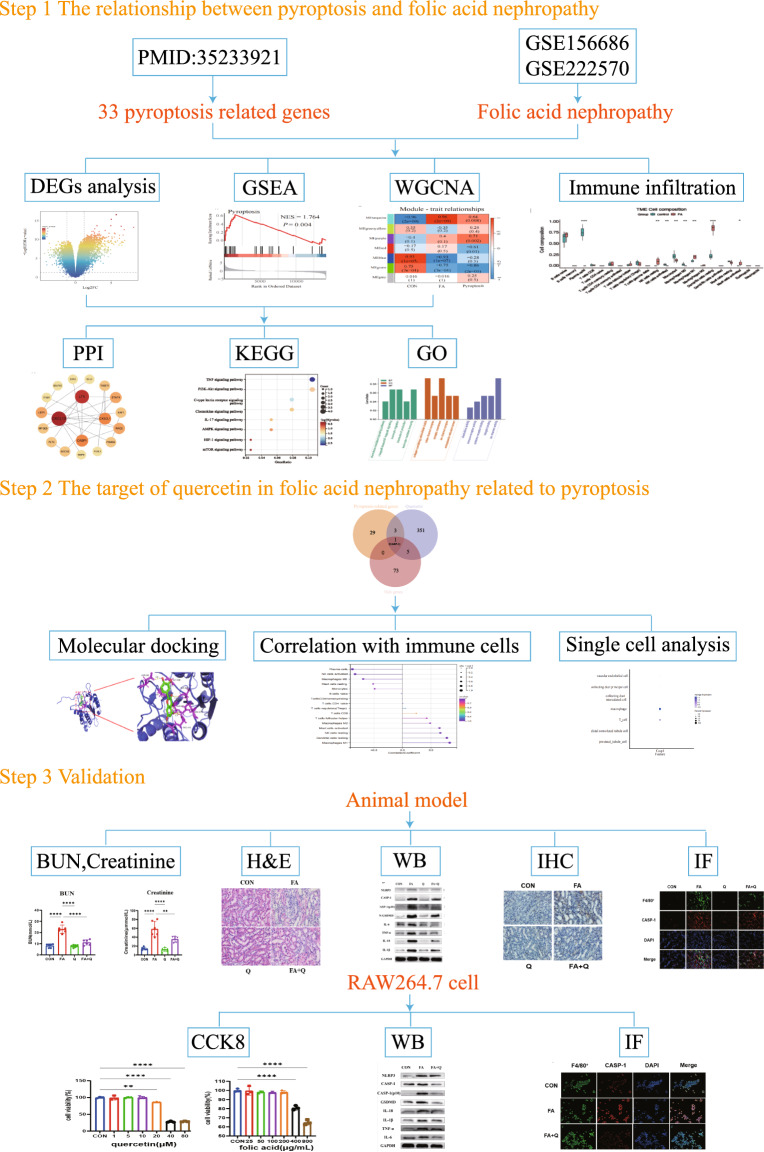
Flowchart of the study

Quercetin, a flavonoid compound found in fruits, vegetables, and plants, demonstrates diverse biological activities encompassing antioxidant [9], anti-inflammatory [10], and anticancer properties [11]. There is accumulating evidence suggesting that quercetin has a potential role against pyroptosis [12–14]. The present study further elucidated the potential mechanisms related to pyroptosis of quercetin ameliorating FA-induced kidney damage. Figure 1 displays a flowchart illustrating the methodology of this study.

## Materials and methods

### Data collection and preprocessing

GSE156686 and GSE222570 were downloaded from the Gene Expression Omnibus (GEO) database (http://www.ncbi.nlm.nih.gov/geo/). The two datasets contain the high throughout sequencing data of nine FA-induced kidney lesion samples and seven control samples in total. To eliminate batch effects, the expression profiles were integrated using the Combat function of the "sva" package.

The structural formula of quercetin was obtained from the PubChem database (https://pubchem.ncbi.nlm.nih.gov/). Quercetin targets were selected from the PharmMapper database (http://www.lilab-ecust.cn/pharmmapper/) and the Swiss TargetPrediction database (http://www.swisstargetprediction.ch/). The data downloaded from the PharmMapper database was converted into the corresponding genes using the UniProt database (https://www.uniprot.org/). The potential drug targets predicted by the two databases, excluding duplicates, were chosen for further verification.

The dataset for single cell RNA sequencing of kidney tissue in the FA-induced renal injury model was also acquired from GSE156686. Downstream analysis was performed using the "Seurat" package.The Mouse Cell Atlas and CellMarker were used to annotate cell types manually.

### Data analysis

Differential expression analysis was conducted on the normalized datasets using the "limma" package (version 3.54.1). We defined differentially expressed genes (DEGs) based on the thresholds of |log2 fold change (FC)|> 1 and *P*.adj value < 0.05.

Thirty-three pyroptosis related genes were obtained from published literature [15]. The enrichment strength was indicated using the normalized enrichment score (NES). The level of significance was defined at *P* < 0.05.

Weighted gene co-expression network analysis (WGCNA) was used to analyze differential genes in the integrated dataset, identifying of coexpression modules and key genes associated with pyroptosis and FA-induced kidney injury. Gene significance (GS) greater than 0.7 and module membership (MM) greater than 0.8 were used to assess the correlation between gene expression and sample trait. Functional enrichment analysis, including Gene Ontology (GO) and Kyoto Encyclopedia of Genes and Genomes (KEGG) pathway analysis, was performed on key module genes using Sangerbox tools (http://sangerbox.com/). Furthermore, WGCNA identified key genes were subjected to protein–protein interaction network analysis using the STRING database (https://cn.string-db.org/) and visualized with Cytoscape software.

The CIBERSORT algorithm was used to assess the presence of immune cell infiltration in kidney tissues based on transcriptome data, with a statistical significance threshold of *P* < 0.05.

The 3D structure of quercetin was obtained from the PubChem database, whereas the protein target was acquired from the Protein Data Bank database. The removal of solvent molecules and ligands was performed using Pymol software, followed by the utilization of AutoDock Tools software to incorporate hydrogen, calculate charges, and assign atomic types. The resulting data was saved in the pdbqt format. Openbel software was used to convert the 3D structure from pdbqt to pdb format. The visualization of specific binding sites and atomic distances between active components and proteins was accomplished using Pymol software.

### Folic acid induced renal kidney model and cell culture

Twenty-four C57BL/6J male mice were randomly assigned to four groups (n = 6): the negative control group (group CON), the FA group (group FA), the quercetin group (group Q), and the quercetin treated with FA group (group FA + Q). The mice in group FA were injected with a single dose of FA (F7876, Sigma-Aldrich) (250 mg/kg, dissolved in 300 mM NaHCO3, i.p.), while the group CON received an equivalent volume of vehicle (300 mM NaHCO3, i.p.). Starting from the day after FA injection, the mice in groups Q and FA + Q were administered quercetin (HY-18085, MCE) at a dosage of 50 mg/kg/day (i.p.). Kidney and blood samples were collected on day 7 after the first injection of FA.

RAW264.7 cells were grown in DMEM supplemented with 10% fetal bovine serum and 1% penicillin/streptomycin and maintained at 37 ℃ in a 5% CO_2_ incubator. Before experimentation, cells were seeded into 6-well plates and incubated overnight. Subsequently, the cells were treated with FA (400 gμ/mL) or a combination of FA (400 gμ/mL) and quercetin (10 mM) for 24 h.

### Cell viability and renal function evaluation

RAW264.7 cells were cultured in 96-well plate and subsequently exposed to varying concentrations of FA (25, 50, 100, 200, 400 and 800 μg/mL) or quercetin (1, 5, 10, 20, 40 and 80 mM). After 2 h of incubation with CCK-8 solution, the optical density at 450 nm was determined to assess cell viability. The obtained results were percentages relative to control and were presented as mean ± standard deviation based on three independent experiments.

According to the instructions provided by the manufacturer, serum creatinine and blood urea nitrogen (BUN) were measured to evaluate renal function.

### Western blots

Using a lysing buffer, we extracted protein from treated RAW264.7 cells or mouse tissue. Protein concentration was determined using the BCA assay. Protein expression levels were assessed through standard western blot procedures, using anti-rabbit primary antibodies targeting NLRP3 (ABclonal, A21906), CASP-1 (ABclonal, A0964), CASP-1(p10) (Abcam, ab179515), IL-18 (ImmunoWay, YN1926), GSDMD (Abcam, ab219800), IL-6 (ABclonal, A0286), TNF-α (ABclonal, A0277), and IL-1β (ABclonal, A16288). A secondary antibody, Goat Anti-Rabbit IgG(H + L)-HRP Conjugate (SAB, L35009), was used. The images were visualized using the Tanon-5200 Chemiluminescent Imaging System, and densitometric analysis was conducted using ImageJ.

### Kidney histology

The kidneys were fixed using a 4% paraformaldehyde solution for a duration of 24 h before paraffin embedding. Sections of the embedded samples were stained with hematoxylin and eosin (H&E) at a thickness of 4 μm. The semiquantitative calculation of the tubular injury score was performed as follows (score 0: no tubular injury; score 1: < 10% tubular injury; score 2: 10–24% tubular injury; score 3: 25–49% tubular injury; score 4: 50–74% tubular damage; score 5: damaged tubules ≥ 75%). Every specimen was subjected to random examination using 400× magnification in ten distinct fields. Six samples were analyzed from each experimental group.

### Immunofluorescence

For immunofluorescence analysis, the renal tissues were embedded in an optimum cutting temperature compound and prepared as frozen sections with a thickness of 5 μm. These sections/cell climbing pieces were fixed with acetone for 10 min and washed with PBS. The sections/pieces were treated with 5% goat serum for 1 h to prevent nonspecific binding. The tissues/macrophages were incubated overnight at 4 ℃ with anti-CASP-1 antibody and anti-F4/80 antibody (Invitrogen, 14-4801-82). The immunofluorescence signals were detected using secondary antibodies, specifically Alexa Fluor 594 donkey anti-rabbit (Abcam, ab150076) and FITC goat anti-rat (Servicebio, GB22302). Additionally, the slides were counterstained with DAPI to visualize the nucleus and subsequently observed under a fluorescent microscope.

### Immunohistochemistry

The paraffin-embedded sections were used for immunohistochemistry staining. Following antigen retrieval, the sections were quenched using a 3% H_2_O_2_ block for 15 min. Incubation with antibodies targeting CASP-1 and F4/80 was performed overnight at 4 °C. Following three washes with PBS, the sections were incubated for 1 h with secondary antibodies that were HRP-labeled. Diaminobenzidine solution was then applied to the slides. The resulting chromogenic reaction, which causes the epitope sites to turn brown, was observed under a microscope. Hematoxylin staining was conducted to counterstain the nuclei.

### Statistical analysis

The data were analyzed using the GraphPad Prism software and presented as mean ± SD/SEM with individual data points as applicable. One-way analysis of variance (ANOVA) with a significance level of *P* < 0.05 was used to assess differences between multiple groups.

## Results

### Screening of differentially expressed genes

**Fig. 2:**
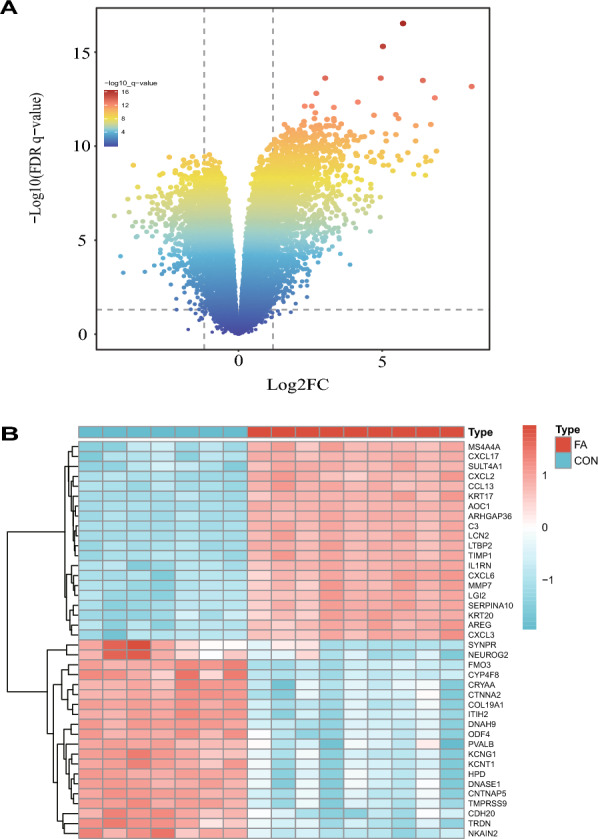
Identification of differentially expressed genes (DEGs) in folic acid nephropathy model. **A** Volcano map of DEGs (|logFC|> 1, *P*.adj < 0.05); **B** Heatmap shows the most significant 20 genes in up- and down-regulation

Based on a significance level of *P*.adj < 0.05 and a threshold of |logFC|> 1, 3068 DEGs were identified, with 1629 upregulated and 1439 downregulated. The volcano plots in Fig. 2A visually represent these DEGs. As displayed in Fig. 2B, heat map exhibited the 20 most significant DEGs among the upregulated and downregulated genes.

### GSEA of pyroptosis and WGCNA of the combined dataset

**Fig. 3:**
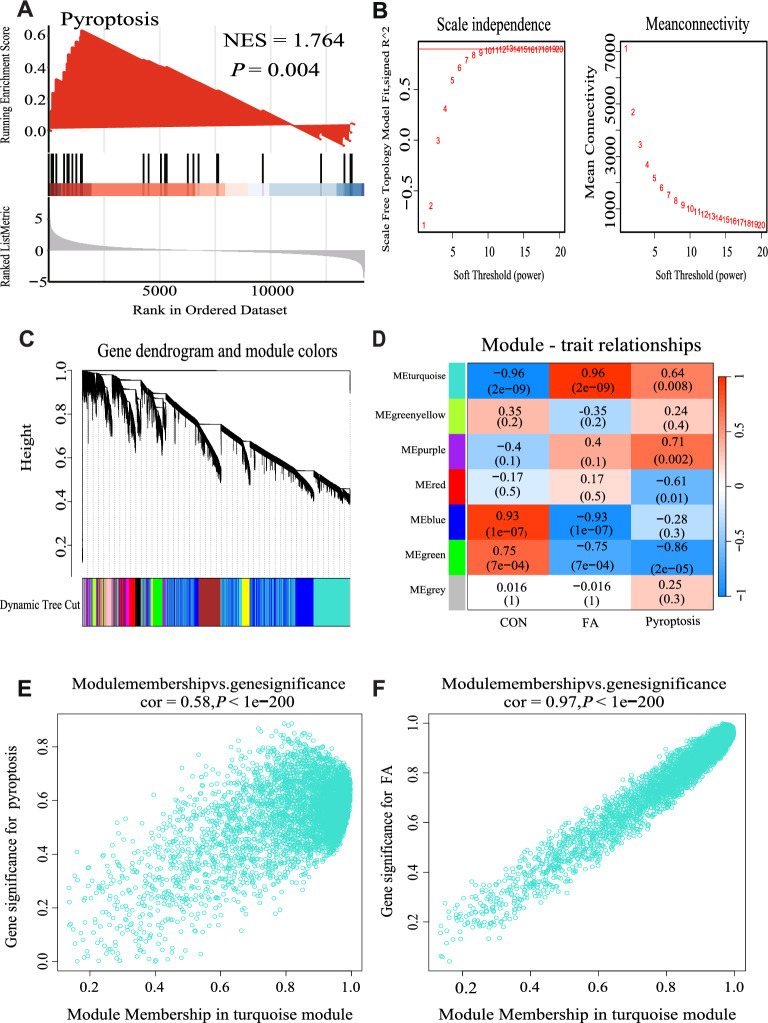
The relationship between pyroptosis and folic acid nephropathy analysed by GSEA and WGCNA. **A** GSEA analysis for pyroptosis in combined dataset; **B** network topology analysis under various soft threshold power; **C** the clustering tree and co-expression network of co-representation network modules are constructed based on 1-TOM matrix; **D** the relationship of three traits and seven modules; **E** the scatterplot describing the relationship between MM and GS in turquoise module related to pyroptosis; **F** the scatterplot describing the relationship between MM and GS in turquoise module related to FA

The FA-induced renal injury was markedly correlated with pyroptosis (NES = 1.764, *P* = 0.004) (Fig. 3A). To build a scale-free network, a soft-threshold of 9 was chosen according to the scale-free topology criterion with an R^2^ value of 0.9 (Fig. 3B). After transforming the adjacency matrix into a TOM matrix, the weighted correlation was used to assess the similarity between nodes. Seven modules were obtained based on average hierarchical clustering and dynamic tree clipping techniques in Fig. 3C and D.

Among these modules, the turquoise module exhibited a strong association with pyroptosis (cor = 0.64, *P* = 0.008) and FA exposure (cor = 0.96, *P* = 2E−09). Consequently, the turquoise module, which is linked to both FA and pyroptosis, was chosen as the hub module for subsequent analysis. The scatter plots illustrating the distribution of genes within the hub module are shown in Fig. 3E and F.

**Fig. 4:**
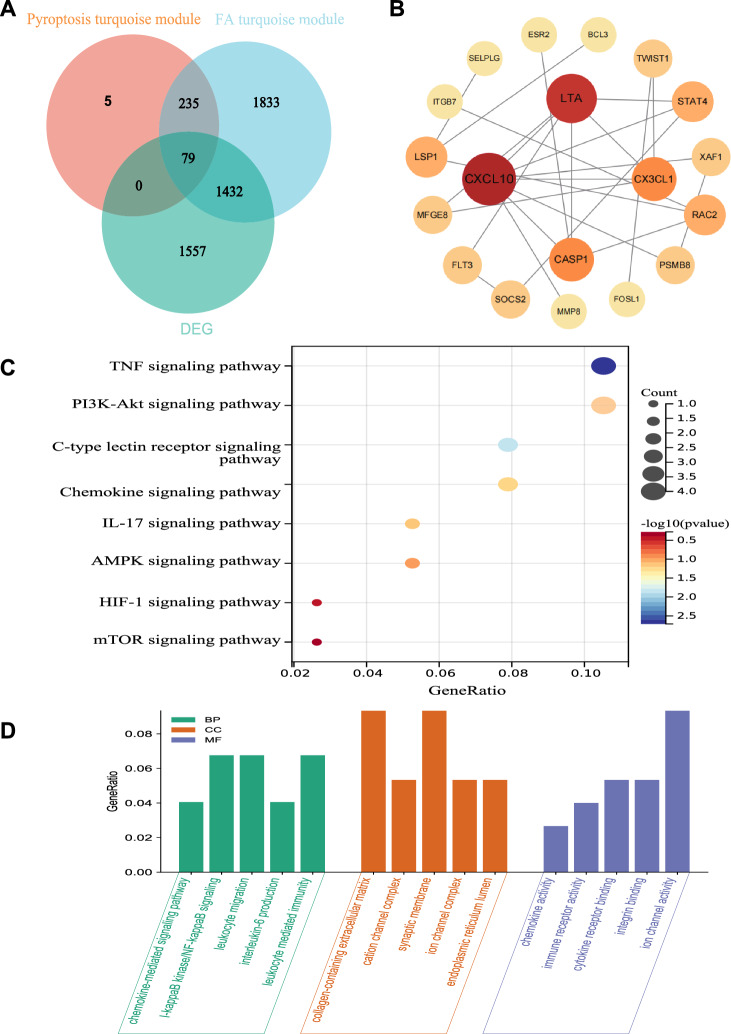
Analysis of hub genes in dataset through WGCNA. **A** Venn diagram showed the intersection genes between module genes and DEGs; **B** PPI network of hub genes; **C** KEGG enrichment of hub genes; **D** GO enrichment of hub genes

The genes exhibiting |GS|> 0.7 and |MM|> 0.8 within each module were subsequently compared to the DEG list, and genes displaying similarity were chosen as the ultimate hub-genes (Fig. 4A). The protein–protein interaction network of the hub genes was assessed using the STRING database and visualized through Cytoscape (Fig. 4B). The outcomes of the KEGG enrichment analysis showed that the genes were primarily found in the TNF signaling pathway, C-type lectin receptor signaling pathway, and IL-17 signaling pathway (Fig. 4C). The GO enrichment analysis revealed that the genes under investigation were significantly associated with chemokine-mediated signaling pathway, leukocyte migration, IL-6 production, and leukocyte mediated immunity (Fig. 4D). These processes are highly interconnected and closely linked to the phenomenon of inflammation.

### Immune cell infiltration results

**Fig. 5:**
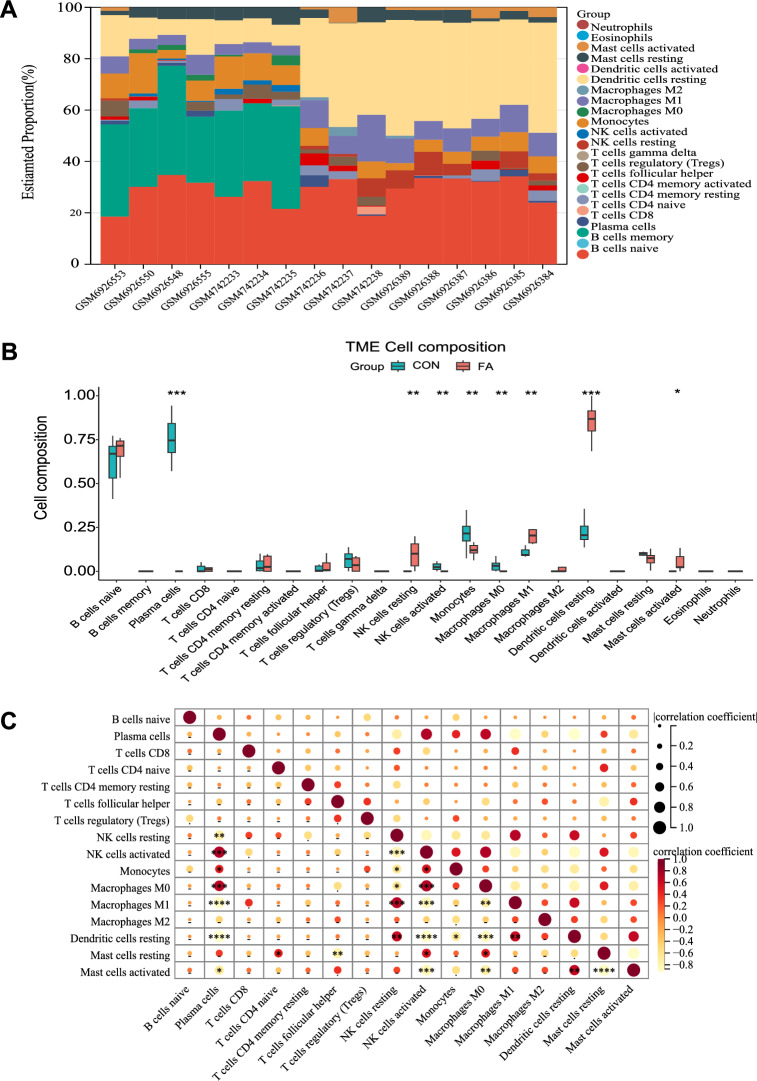
Immune infiltration landscape in folic acid nephropathy. **A** Heat map of relative proportions of 22 infiltrated immune cells; **B** comparisons of immune cells between folic acid induced kidney injury and control; **C** the co-expression patterns among fractions of immune cells

The heatmap displayed the disparity in immune cell infiltration between two groups (Fig. 5A). The violin plot demonstrated that, in comparison to the control sample, NK cells resting, macrophages M1, dendritic cells resting, and activated mast cells exhibited higher levels of infiltration (Fig. 5B). Furthermore, an analysis of correlation was conducted to examine the connection between infiltrated immune cells, revealing several pairs of positively and negatively correlated immune cells (Fig. 5C).

### The target of quercetin alleviated kidney injury related to pyroptosis

**Fig. 6:**
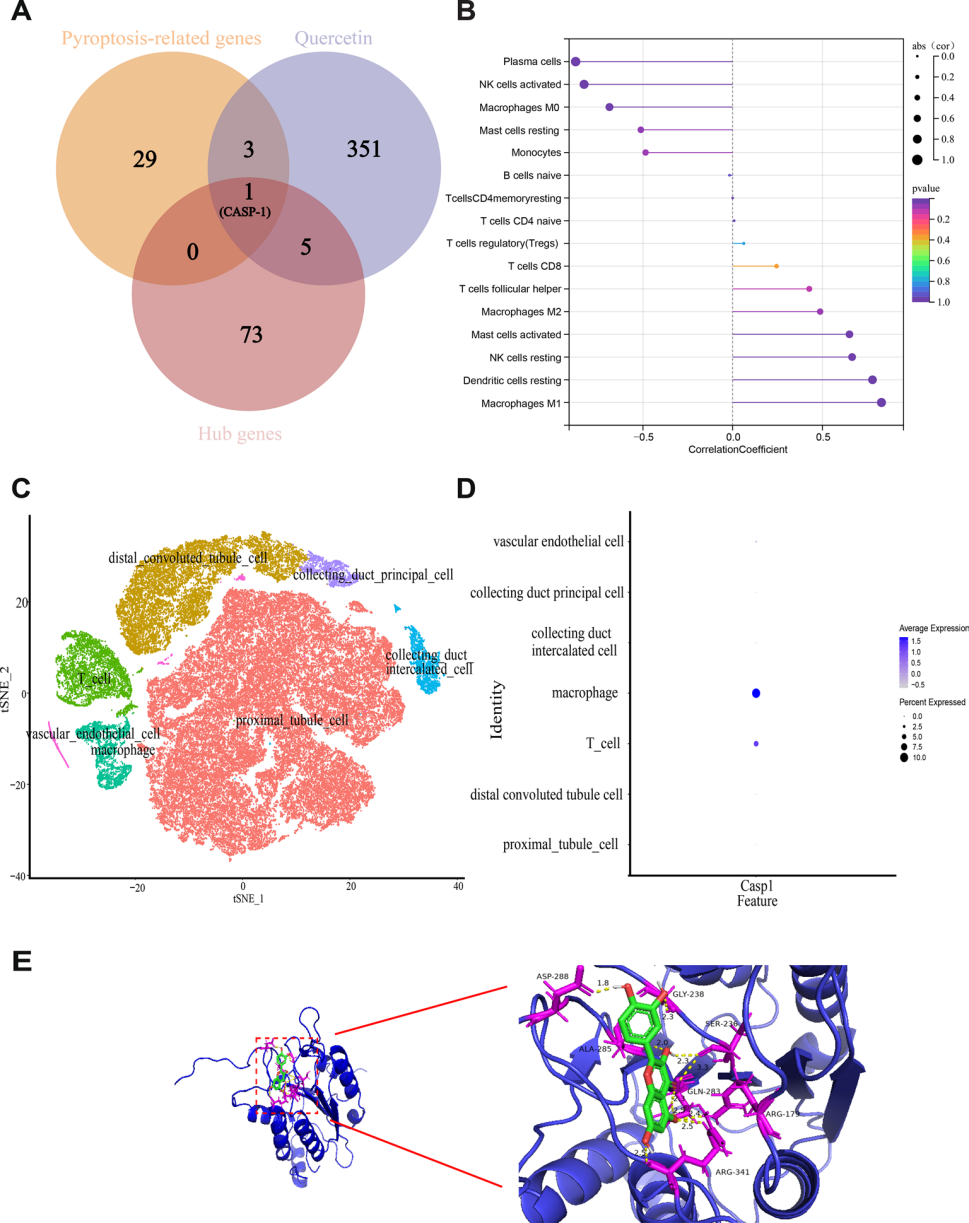
Identify the target of quercetin related to pyroptosis. **A** Venn diagram showed the target of quercetin related to pyroptosis in FA animal model; **B** the relationship between CASP-1 expression and immune cell infiltration level; **C** cell subset annotation of renal tissue; **D** expression pattern of CASP-1 at the single cell level; **E** molecular docking of CASP-1 and quercetin

360 target genes of quercetin (Additional file 1: Table S1) were gained. By identifying the hub genes within the module and their intersection with pyroptosis-related genes, it was determined that CASP-1 may serve as a potential target for quercetin in mitigating pyroptosis-induced kidney injury (Fig. 6A).

To examine the relationship between the CASP-1 gene and immune cells, we analyzed the expression data of CASP-1 in the combined dataset. A positive correlation was found between the expression value of CASP-1 and the levels of infiltration of macrophages M1, dendritic cells resting, NK cells resting, and mast cells activated. The strongest correlation was observed with macrophages M1 (R = 0.82) (Fig. 6B). To perform unsupervised cell clustering analysis, we used t-distributed random neighborhood embedding (t-SNE). Figure 6C demonstrates the segregation of renal tissue into seven distinct clusters. Subsequently, the expression pattern of CASP-1 in the aforementioned cell clusters was examined, and its expression at the single-cell level was confirmed (Fig. 6D). Notably, the macrophages exhibited a significantly elevated expression of CASP-1. The binding energy of − 6.49 kcal/mol suggests a favorable binding affinity between quercetin and CASP-1 (Fig. 6E).

### Quercetin alleviated kidney injury of folic acid animal model

**Fig. 7:**
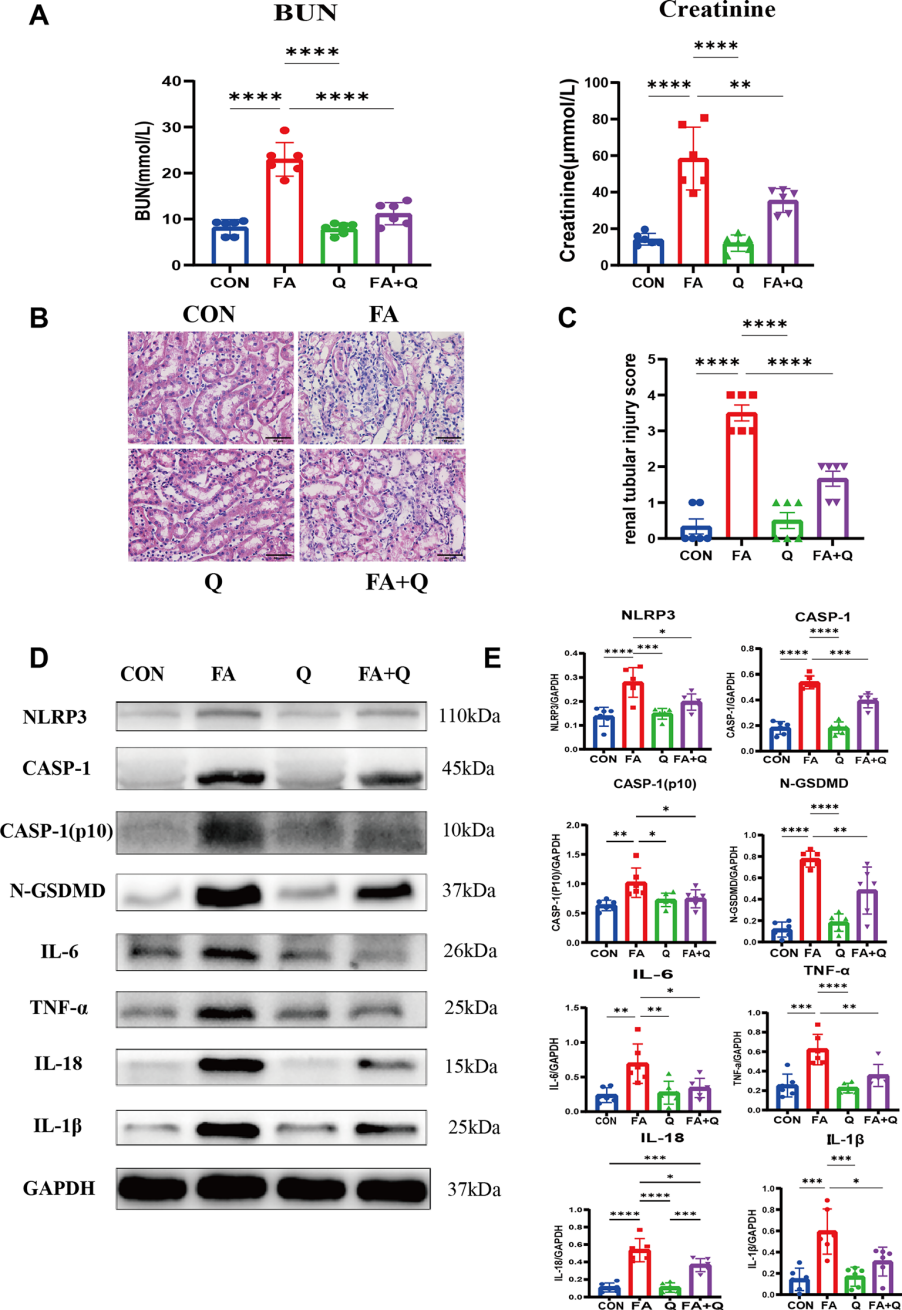
Quercetin alleviates kidney injury and inhibits pyroptosis in folic acid nephropathy model. **A** Serum level of BUN and creatinine; **B** H&E staining showed quercetin inhibited kidney injury induced by FA; **C** the renal tubular injury score of different groups, data represent mean ± SEM (n = 6); **D** Western blot revealed quercetin attenuated protein expression related pyroptosis in FA animal model; **E** quantitative analysis of western blot, data represent mean ± SD (n = 6). Scale bar = 50 μm, *p < 0.05, **p < 0.01, ***p < 0.001, ****p < 0.0001

**Fig. 8:**
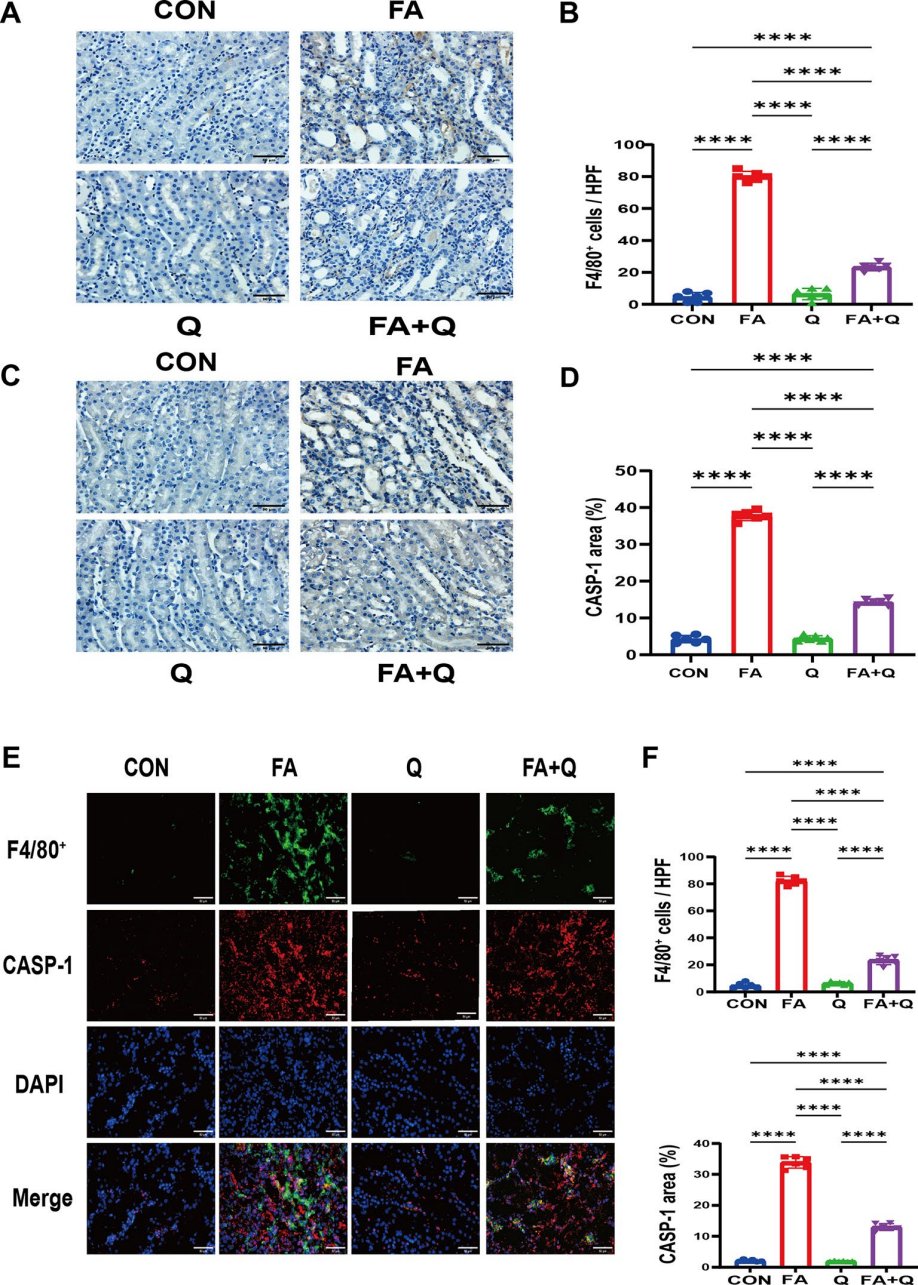
Quercetin inhibits macrophage infiltration and CASP-1 expression in vivo. **A** Representative Immunohistochemical images of F4/80^+^ cells in kidney sections; **B** quantitative analysis of F4/80^+^ positive cells in the kidneys; **C** representative Immunohistochemical images of CASP-1 in kidney sections; **D** quantitative analysis of CASP-1 positive area in the kidneys; **E** representative photomicrographs showing the immunofluorescence staining for F4/80^+^ (green) and CASP-1 (red) among different groups; **F** quantitative analysis of F4/80^+^ positive cells and CASP-1 positive area in kidney sections measured by immunofluorescence staining. Data represent mean ± SD (n = 6). Scale bar = 50 μm, ****p < 0.0001

Quercetin did not exhibit any discernible effect on normal mice. However, it demonstrated a protective function by diminishing the concentrations of BUN and serum creatinine (Fig. 7A). The evaluation of tubular injury in kidney sections through H&E staining revealed that quercetin significantly mitigated histologic injury (Fig. 7B). Western blot analysis of the kidney exhibited elevated expression levels of NLRP3, CASP-1, CASP-1(p10), N-GSDMD, IL-18, IL-1β, IL-6, and TNF-α in group FA, which were subsequently normalized by the administration of quercetin (Fig. 7D). There was a higher CASP-1 and F4/80 positive area in group FA compared to groups CON and Q. While the effect was attenuated by the administration of quercetin (Fig. 8A, C). To further investigate the type of macrophage death in the kidneys and the potential mechanism of quercetin’s protective effect against renal injury, CASP-1 and F4/80 double staining was performed to indicate the co-localization of CASP-1 in macrophages (Fig. 8E). These findings suggested that macrophage pyroptosis is a characteristic of FA-induced renal injury and that quercetin can suppress macrophage pyroptosis.

### Quercetin inhibited RAW264.7 pyroptosis

**Fig. 9:**
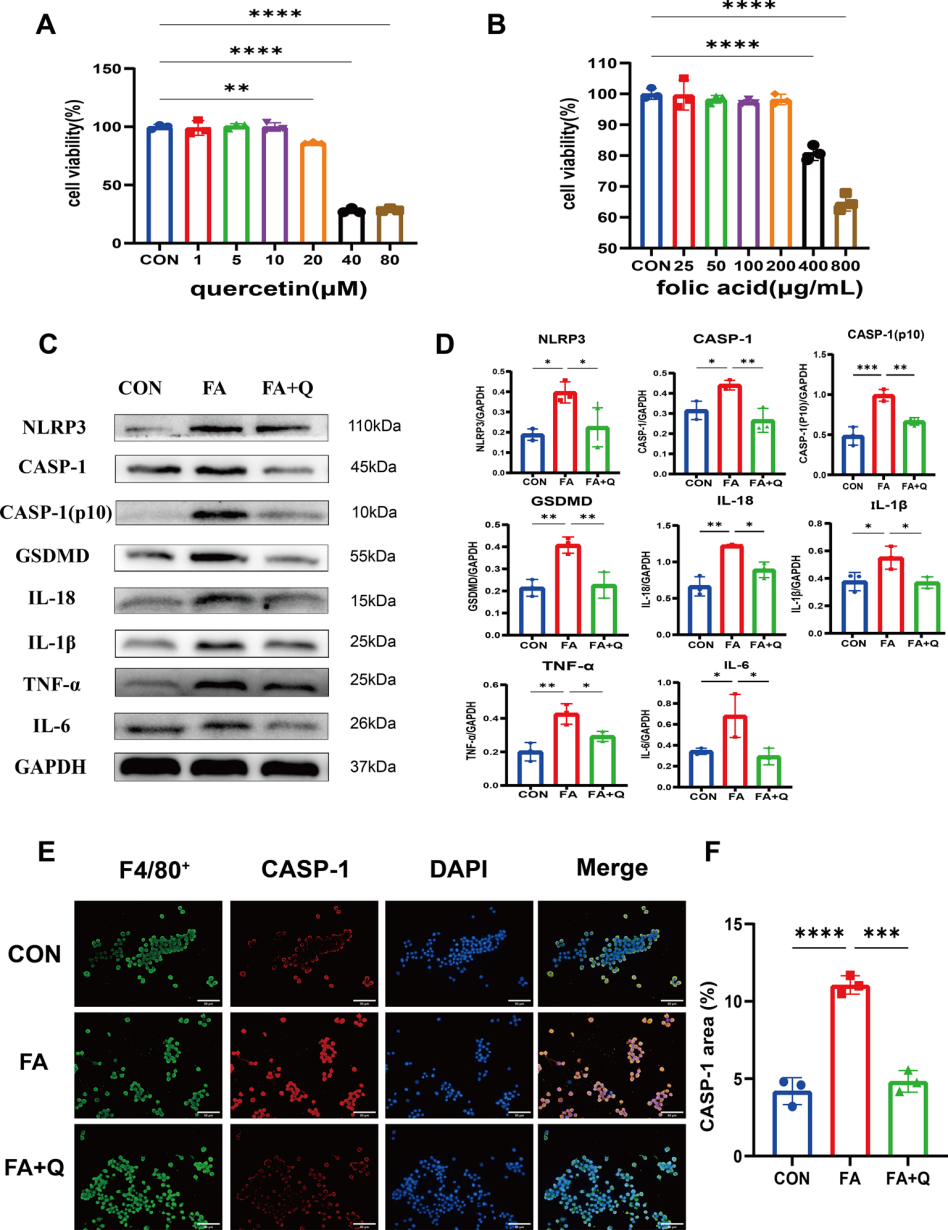
Quercetin inhibits CASP-1 expression and related pyroptosis in RAW264.7 cells. **A** Cytotoxic assessment of quercetin in RAW264.7 cells; **B** cytotoxic assessment of folic acid in RAW264.7 cells; **C**, **D** immunoblots and quantitative histograms showing the results of Western blot analysis for the expression of protein related to pyroptosis; **E** representative microscopy images of cells stained for F4/80^+^ (green), CASP-1 (red) and nuclei (blue) to detect expression of CASP-1 (Scale bars: 50 μm); **F** quantitative analysis of CASP-1 positive area in cells. Data represent mean ± SD (n = 3). *p < 0.05, **p < 0.01, ***p < 0.001, ****p < 0.0001

At concentrations of quercetin 10 μM /FA 200 gµ/mL or lower, there was no observed effect on the RAW 264.7 cell viability (Fig. 9A, B). Exposure to FA increased in the expression levels of pyroptosis-related proteins NLRP3, CASP-1, CASP-1(p10) and GSDMD, subsequently promoting the production of pro-inflammatory mediators such as Il-1β, IL-18, IL-6, and TNF-α (Fig. 9C). However, quercetin significantly inhibited the expression of these proteins and the production of pro-inflammatory mediators. To further assess the effect of FA on macrophages, the expression of CASP-1 was measured by co-staining with the macrophage marker F4/80. The results demonstrated a significant increase in intracellular CASP-1 fluorescence intensity upon exposure to FA, which was significantly suppressed by treatment with quercetin (Fig. 9E).

## Discussion

Pyroptosis has been documented in diverse inflammatory kidney disorders involving acute kidney injury [16], chronic kidney disease [7], and diabetic nephropathy [17]. The mechanisms underlying pyroptosis in tubular epithelial cells involve Caspase-11/GSDMD [18], Caspase-3/GSDME [19], and miR-342-3p/CASP-1 [20]. Caspase-11/4 and GSDMD have been found to contribute to podocyte pyroptosis in diabetic nephropathy [21]. Vascular smooth muscle cell pyroptosis was also identified in adenine-induced CKD mice [7]. These findings highlight the relevance of pyroptosis in kidney disease. Our study used GSEA and WGCNA analysis to unveil further the substantial involvement of pyroptosis in the advancement of kidney injury. As a highly pro-inflammatory form of cell death, pyroptosis exhibits a close association with immune cell infiltration [22], which is recognized as a crucial mechanism contributing to kidney injury. As shown in our study, the enrichment analysis of the identified hub genes demonstrated their predominant participation in inflammatory pathways and processes.

CASP-1, an inflammatory caspase, is critical in the canonical inflammasome-mediated pyroptosis and cytokine maturation. Upon activation, CASP-1 cleaves GSDMD, an effector molecule involved in pyroptosis. The cleaved form of GSDMD then induces the formation of pores in the cell membrane, causing cellular swelling and rupture, ultimately releasing pro-inflammatory cytokines and cell debris. Several studies have suggested a potential association between CASP-1 activation and conditions such as ischemia–reperfusion injury [23], diabetic nephropathy [24], and the progression of renal fibrosis [7]. VX-765, a specific inhibitor targeting CASP-1, demonstrated a mitigating effect on pyroptosis in both ketamine-induced renal injury [25] and diabetes [26]. Despite the validation of various CASP-1 inhibitors in animal models of kidney disease [27, 28], their utilization in humans is constrained due to their unfavorable toxicity profiles.

Quercetin, a dietary compound, has been found to possess potential protective properties against kidney disease [29, 30]. Its ability to suppress inflammasome activation [31] and regulate pro-inflammatory cytokines [32] suggests that it may have the potential to mitigate pyroptotic cell death. Quercetin mitigated the neurotoxicity induced by multi-walled carbon nanotubes by inhibiting pyroptosis in neuron cells [33]. Additionally, quercetin has demonstrated protective effects against intestinal inflammation [34], osteolysis [35], steatohepatitis [36] and LPS-induced lung injury [37] by inhibiting pyroptosis. Moreover, quercetin ameliorated HK-2 cell pyroptosis in lupus nephritis [38]. Through molecular docking and single-cell analysis, the target of quercetin was identified as CASP-1, which is mainly expressed in macrophages. The present study revealed a significant reduction in CASP-1 levels in macrophages following treatment with quercetin. However, the NLRP3 inflammasome, which serves as the primary mediator for CASP-1 activation, was also mitigated by quercetin. This observation implies that additional mechanisms may be involved in the regulation of quercetin. Competitive binding to KEAP1 via Arg483 to inhibit macrophage pyroptosis has been demonstrated in the protection of quercetin anti-atherosclerosis [12]. There is evidence showed that quercetin inhibited THP-1 macrophage pyroptosis induced by LPS/ATP treatment via the TLR2/Myd88/NF-κB and ROS/AMPK pathways [39]. Further investigations are warranted to comprehensively understand the mechanisms and efficacy of quercetin in preventing or ameliorating pyroptosis-related disorders.

## Conclusions

**Fig. 10:**
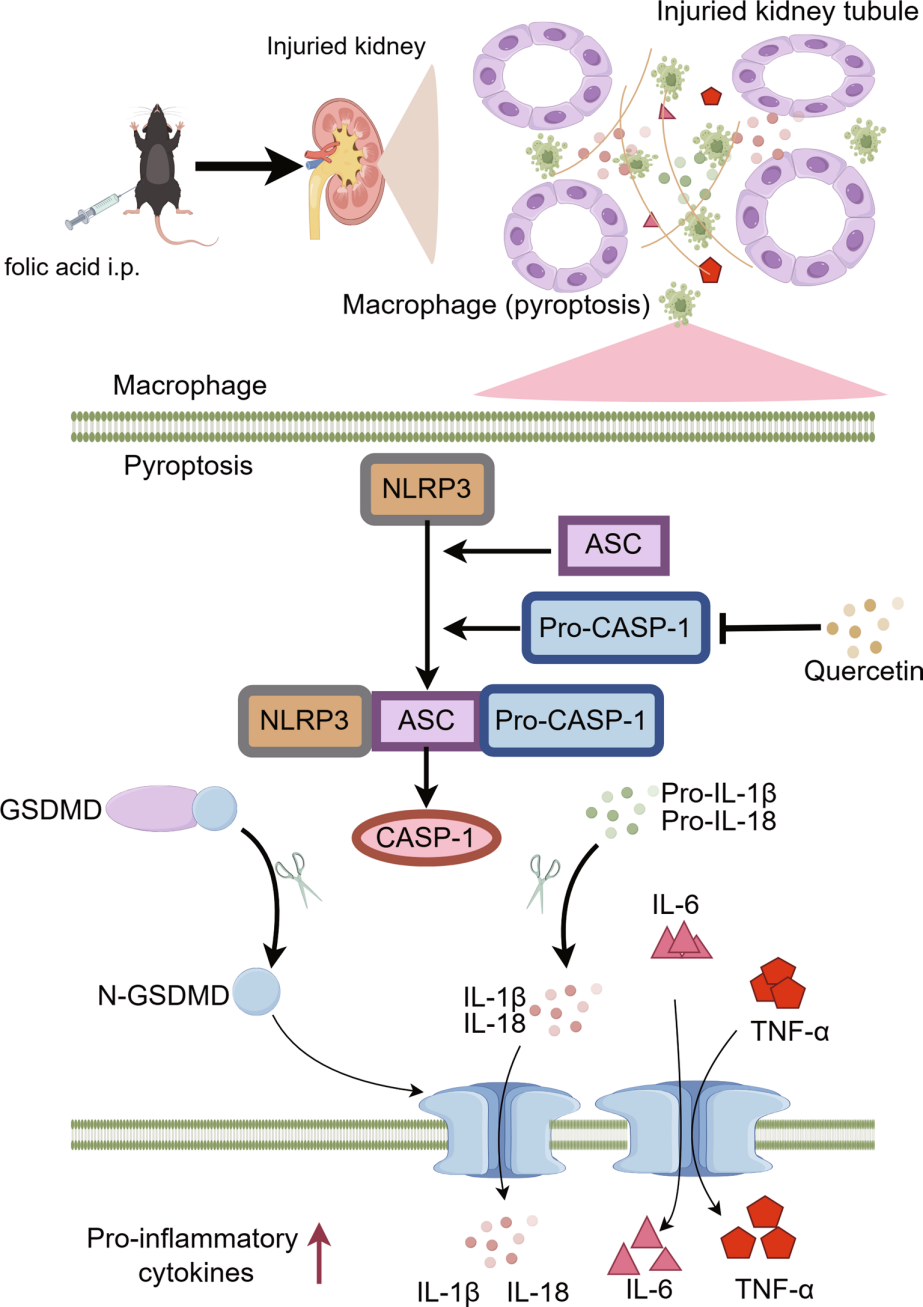
Hypothesis mechanism map was drawn by Figdraw

The present study proves that pyroptosis plays a significant role in the advancement of kidney injury induced by high doses of folic acid (Fig. 10). Quercetin efficiently reduced the expression of CASP-1 within macrophages, thereby alleviating pyroptosis.

### Supplementary Information

**Additional file 1.** Target genes of quercetin.

## Data Availability

All the data used to support the findings of this study are available from the corresponding author upon reasonable request.

## Abbreviations

GSEA: Gene set enrichment analysis
WGCNA: Weighted gene co-expression network analysis
FA: Folic acid
CASP-1: Caspase-1
GEO: Gene Expression Omnibus
NES: Normalized enrichment score
DEG: Differentially expressed gene
GO: Gene Ontology
KEGG: Kyoto Encyclopedia of Genes and Genomes
NLRP3: NACHT, LRR and PYD domains-containing protein 3
GSDMD: Gasdermin-D
N-GSDMD: N terminal of GSDMD
IL: Interleukin
TNF: Tumor necrosis factor
